# Efficacy of Ventilation Tube Insertion with Palatal Repair for Otitis Media in Cleft Palate: Meta-Analysis and Trial Sequential Analysis

**DOI:** 10.3390/jpm12020255

**Published:** 2022-02-10

**Authors:** Feng-Liang Chang, Chih-Hao Chen, Hsiu-Lien Cheng, Chun-Yu Chang, Jing-Li Leong, Yen-Ting Chang, Yen-Fu Cheng, Wen-Huei Liao

**Affiliations:** 1 Department of Otolaryngology—Head and Neck Surgery, Taipei Veterans General Hospital, Taipei 112, Taiwan; harry14harry14@gmail.com (F.-L.C.); michaelchen808@gmail.com (C.-H.C.); hlcheng@vghtpe.gov.tw (H.-L.C.); 2 Department of Biomedical Engineering, National Yang Ming Chiao Tung University, Taipei 112, Taiwan; 3 Department of Anesthesiology, Taipei Tzu Chi Hospital, Buddhist Tzu Chi Medical Foundation, New Taipei City 231, Taiwan; paulchang1231@gmail.com; 4 Department of Medical Education, Taipei Veterans General Hospital, Taipei 112, Taiwan; leongjingli@gmail.com; 5 Department of Medical Education, Taipei Tzu Chi Hospital, Buddhist Tzu Chi Medical Foundation, New Taipei City 231, Taiwan; 103311111@gms.tcu.edu.tw; 6 Department of Medical Research, Taipei Veterans General Hospital, Taipei 112, Taiwan; 7 Faculty of Medicine, National Yang Ming Chiao Tung University, Taipei 112, Taiwan; 8 Institute of Brain Science, National Yang Ming Chiao Tung University, Taipei 112, Taiwan

**Keywords:** cleft palate, otitis media with effusion, ventilation tube insertion, palatal repair

## Abstract

Cleft palate is the most common congenital facial deformity and may result in multiple sequelae and disabilities. One common comorbidity is refractory otitis media with effusion (OME), as patients with cleft palate have impaired eustachian tube function with alteration of the nearby muscular structures. Ventilation tube insertion (VTI) is regarded as an effective mean to address OME in addition to palatal repair surgery. However, controversy regarding the efficacy of VTI and the timing of VTI remains. We aimed to assess the efficacy of VTI with palatal repair for cleft palate on OME development via a meta-analysis with systematic review and trial sequential analysis (TSA). Studies including patients with cleft palate who underwent palatal repair with or without VTI were considered eligible. After searching the Cochrane Library, PubMed, EMBASE, Web of Science, Scopus and China National Knowledge Infrastructure (CNKI) from inception through 5 September 2021, 9 studies involving 929 patients were included. Overall, a significantly higher OME-free rate was noted in those who underwent VTI and palatal repair than in those who underwent palatal repair alone (OR, 2.73; 95% CI, 1.37 to 5.42; *p* = 0.004; *I*^2^ = 84%). Subgroup analysis revealed that the OME-free rate remained higher in the concurrent VTI group (OR, 3.29; 95% CI, 1.64 to 6.59; *p* < 0.001; *I*^2^ = 81%). TSA indicated that all the analyses provided conclusive results by meeting the required information size and Z-value. The meta-analysis indicated that VTI is an effective procedure to prevent OME in patients with cleft palate and that VTI is beneficial when performed concurrently with palatal repair surgery.

## 1. Introduction

Cleft lip and palate are the most common congenital facial deformities. The main cause is malformation of the central prominence and left and right maxillary prominences during embryonic development. According to the U.S. Centers for Disease Control (CDC), approximately 1 in every 1600 babies is born with cleft lip and palate in the United States, and isolated cleft palate accounts for 1 in every 1700 babies [1]. Malformation of the palate causes both cosmetic and functional problems. As the cleft palate affects the nearby muscular structures, including the tensor veli palatini muscle and levator veli muscle, abnormal contraction of these structures results in impaired eustachian tube function, mainly impaired opening function. Consequently, ventilation of the middle ear is disturbed, and otitis media with effusion (OME) develops easily. Approximately 90% of infants with cleft palate develop OME at birth [2,3]. Although OME is a common disease in pediatric populations, it is not self-limited in patients with cleft palate unless further intervention is performed. Subsequent complications, including permanent hearing loss, could be devastating, as they would affect the patient’s quality of life and speech and communication ability, which would ultimately lead to psychosocial problems [4]. Studies have indicated that patients with cleft palate tend to have speech problems and impaired social function [4,5,6].

Over the past several decades, great knowledge and breakthroughs in surgical techniques regarding cleft palate have been gained. The disease is no longer unmanageable with the cooperation of a multidisciplinary team, including a pediatric otolaryngologist, plastic surgeon and speech therapist [4,5,6,7]. Various palatal repair procedures have been developed, all with inspiring results in terms of cosmetic and functional outcomes [7,8]. However, the ventilation function of the middle ear does not show proportional recovery in accordance with the other outcomes after palatal repair, since some patients with cleft palate undergoing palatal repair still develop OME [3,9]. Under these circumstances, ventilation tube insertion (VTI) has been regarded as a potential means to cope with OME, as suggested by the American Academy of Otolaryngology—Head and Neck Surgery Foundation (AAO-HNSF), in children with cleft palate, who are at a high risk for complicated OME and may need further VTI [10,11,12].

By placing the ventilation tube, the middle-ear cavity is able to communicate with the external environment to eliminate the negative pressure caused by the absorption of gases in the middle ear, and the accumulated effusion can therefore be drained out from the Eustachian tube, which can further prevent inflammation behind the eardrum and brake the vicious cycle of OME formation [3,13,14]. However, whether VTI can truly help patients with cleft palate remains controversial [10,15]. Though there is much development in the design and material of ventilation tubes, including biocompatible tubes and substance-coated tubes, which provides diversified choice of tubes according to clinical requirement [16,17,18], VTI itself still cause various complications, including secondary infection or tube-related problems (e.g., tube displacement or prolonged indwelling) and the best timing of VTI is unclear [10,19]; clinicians may choose to perform VTI concurrently with palatal repair surgery or to postpone VTI until OME develops [10,15]. Different policies for VTI are adopted across different countries, and it remains inconclusive whether VTI is effective for the management of OME in patients with cleft palate. Under these circumstances, this study aims to provide comprehensive evidence on the efficacy of VTI with palatal repair in the prevention of OME by a systematic review and meta-analysis.

## 2. Materials and Methods

### 2.1. Study Design

The present study is a systematic review with meta-analysis following the Preferred Reporting Items for Systematic Reviews and Meta-Analyses (PRISMA) guidelines [20].

### 2.2. Search Strategy

From inception through 5 September 2021, databases including the Cochrane Library, PubMed, EMBASE, Web of Science, Scopus and China National Knowledge Infrastructure (CNKI) were searched. We used a combination of medical subject headings (MeSH) and text words to create three citation subsets: one included studies on populations with cleft palate (“Cleft”, “Palate cleft”), one included studies on intervention with VTI (“Ventilation tube” OR “Grommet tube” OR “Tympanostomy tube insertion”), and one included studies on specific outcome evaluations (“Otitis media” OR “Middle ear effusion” OR “Secretory otitis media” OR “Hearing loss”). The detailed search strategy is shown in Table S1 in the Supplementary Materials. The titles, abstracts, and keywords of identified records were screened. The full texts of eligible records were then reviewed.

### 2.3. Eligibility Criteria

After review by two authors (F.-L. Chang and C.-H. Chen), the effect estimates of interest were extracted. Primary data were analyzed to evaluate whether they met all of the following criteria: (a) the study enrolled patients with a palatal cleft who had OME and underwent palatal repair surgery; (b) the study allocated patients to undergo an intervention with or without VTI; and (c) the study provided adequate information and outcomes of interest (e.g., the rate of patients free from OME) to calculate the effect estimates for meta-analysis. We did not exclude studies based on publication date, language, or geographical area. If there were discrepancies regarding the inclusion of a study, a third author (W.-H. Liao) was consulted for a consensus or discussion. The OME-free rate was defined by a normal tympanogram (e.g., type A) or by direct observation by an experienced doctor confirming the absence of OME. Post-VTI otorrhea was extracted to evaluate the safety of VTI in patients with cleft palate.

### 2.4. Risk of Bias Assessment

The Risk of Bias in Nonrandomized Studies of Interventions (ROBINS-I) tool [21] was used to assess the methodological quality of the included studies. Disagreements were resolved by a third responsible author (W.-H. Liao).

### 2.5. Statistical Analysis

The random-effects model was used for effect size calculation under the assumption that a second source of error other than sampling error existed. Statistical heterogeneity was assessed by the Cochran Q test and the I2 statistic. Heterogeneity was regarded as low, moderate, and high at I2 values of <50%, 50–74%, and ≥75%, respectively [22]. Subgroup analyses were performed to explore the influence of the timing of VTI, as it may influence the environment of the middle ear. The influence analysis of the OME-free rate was performed with the pooled point estimates by omitting one included study at a time. Additionally, trial sequential analysis (TSA) was performed to evaluate whether the result was subject to type I or type II error caused by a lack of data or power. The traditional significance boundary in TSA analysis of −1.96 to 1.96 was used, and the sequential monitoring boundary varied by analysis. For the testing of futility, if the cumulative Z-curve fell in the futility boundary or the inner wedge of futility, a nonsignificant result would be confirmed [23,24]. The models for all outcomes were assessed considering an alpha value of 0.05 and a power of 80%. Finally, publication bias was evaluated for the results by a contour-enhanced funnel plot [25,26] with Egger’s test to assess the asymmetry [27]. All of the calculations for the meta-analysis were performed in R studio with the metaphor package [28], and the TSA was performed using TSA software version 0.9.5.10 Beta [23,24].

## 3. Results

### 3.1. Study Identification and Selection

**Figure 1:**
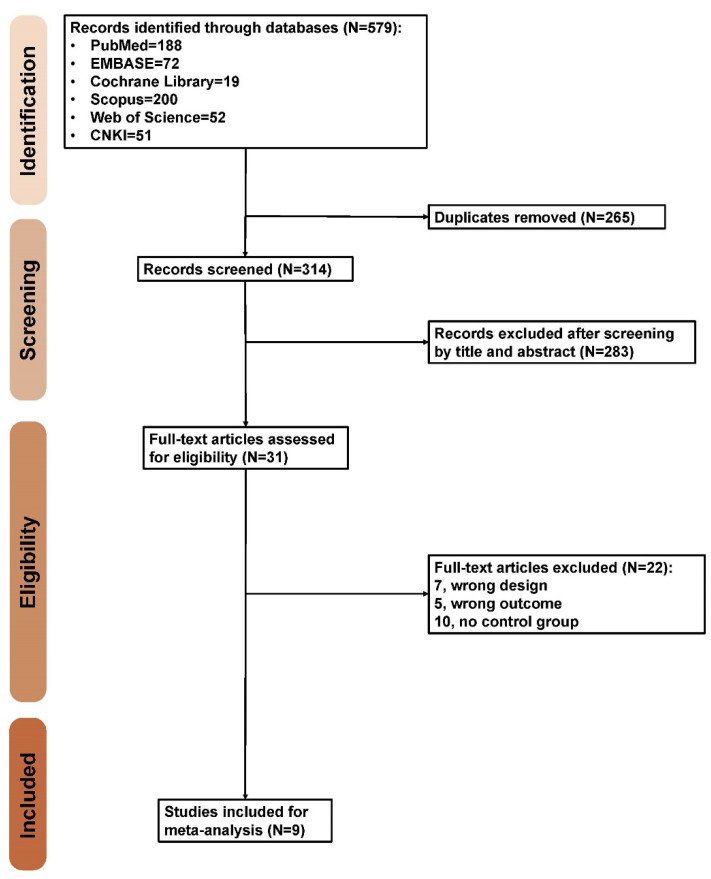
Preferred Reporting Items for Systematic Reviews and Meta-Analyses (PRISMA) flow diagram.

The present study identified 579 records in the preliminary search. After removing duplicates and screening titles and abstracts, 31 studies eventually underwent full-text review. Twenty-two studies were excluded due to a lack of comparison to the control group (N = 10), an inappropriate study design (N = 7) or an inappropriate outcome (N = 5). As a result, 9 eligible studies were included (Figure 1).

### 3.2. Study Characteristics and Risk-of-Bias Assessment

A total of 929 patients were allocated into the group of palatal repair with VTI and the group with palatal repair without VTI. All of the studies were composed of patients with cleft palate. Eight of the included studies enrolled patients who underwent VTI concurrently during palatal repair [29,30,31,32,33,34,35,36], while one of them included patients who underwent VTI after palatal repair [37]. Six studies assessed the OME status by tympanography [30,31,32,33,34,37], and the other two studies evaluated the presence of OME by both otoscopy and tympanography [35,36]. Five included studies reported the post-VTI otorrhea rate and were further analyzed via a meta-analytic procedure [29,30,31,33,37]. Detailed information is presented in Table 1.

Risk of bias was assessed for each of the included studies. All the studies were categorized as having moderate to serious bias due to potential cofounding factors [29,30,31,32,33,34,35,36,37]. Moderate bias due to the selection of participants existed in one study, as the enrollment did not declare clearly [37]. Two included studies exhibited moderate bias in the classification of interventions [30,36]. Serious bias due to missing data was present in one included study because some patients did not adhere to the follow-up schedule [29]. Five studies exhibited moderate bias in the measurement of outcomes [29,31,36,37]. Finally, bias in selection of the reported results was present in four included studies [29,30,31,37]. The detailed assessment is presented in the Supplementary Materials (Figures S1 and S2).

### 3.3. Outcomes

#### 3.3.1. OME Prevention

**Figure 2:**
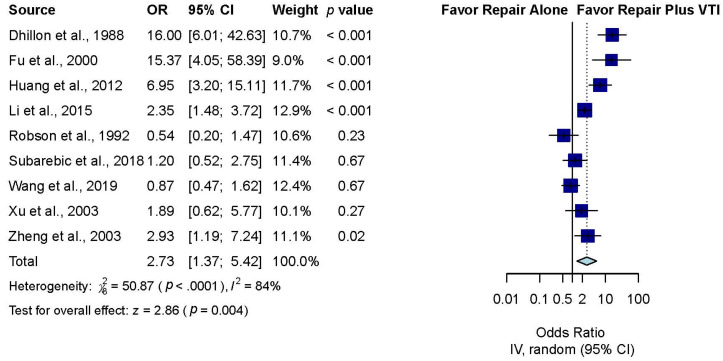
Overall effect of VTI with palatal repair on the otitis media with effusion (OME)-free rate.

Eleven studies compared the OME-free rate among included patients between palatal repair with and without VTI [29,30,31,32,33,34,35,36,37,38,39]. Overall, the pooled result revealed a significantly higher OME-free rate after palatal repair plus VTI than after palatal repair alone (OR, 2.73; 95% CI, 1.37 to 5.42; *p* = 0.004; *I*^2^ = 84%) (Figure 2).

#### 3.3.2. Subgroup Analysis by the Timing of VTI

**Figure 3:**
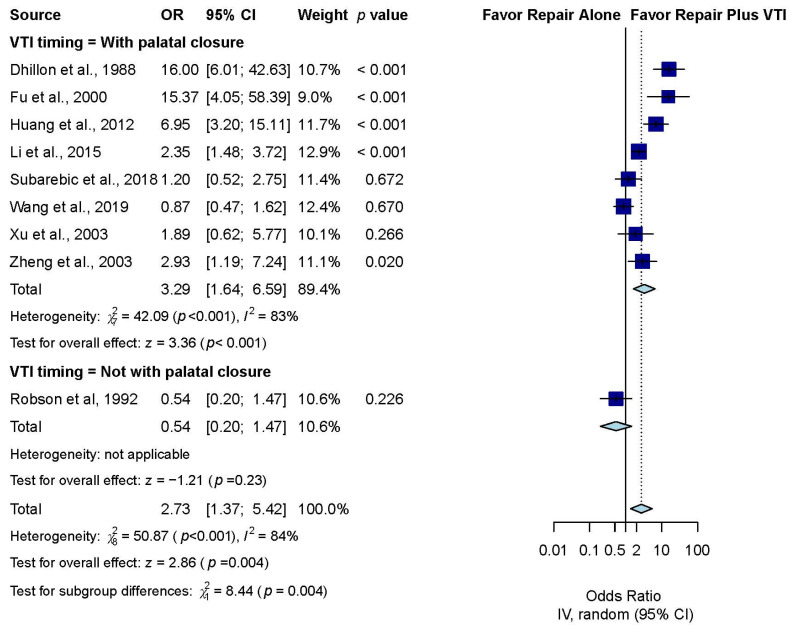
Subgroup analysis of OME-free rate by VTI timing.

Subgroup analysis was performed according to the timing of VTI. Nine included studies [29,30,31,32,33,34,35,36,38] enrolled patients who underwent concurrent VTI with palatal repair, and the pooled result showed a significantly higher OME-free rate in these patients than in patients undergoing palatal repair without VTI (OR, 3.29; 95% CI, 1.64 to 6.59; *p* < 0.001; *I*^2^ = 81%). Another one included studies enrolled patients who underwent VTI after palatal repair [29] and the result showed no significant difference between the intervention and control groups (OR, 0.54; 95% CI, 0.20 to 1.47; *p* = 0.226) (Figure 3).

#### 3.3.3. Post-VTI Otorrhea

**Figure 4:**
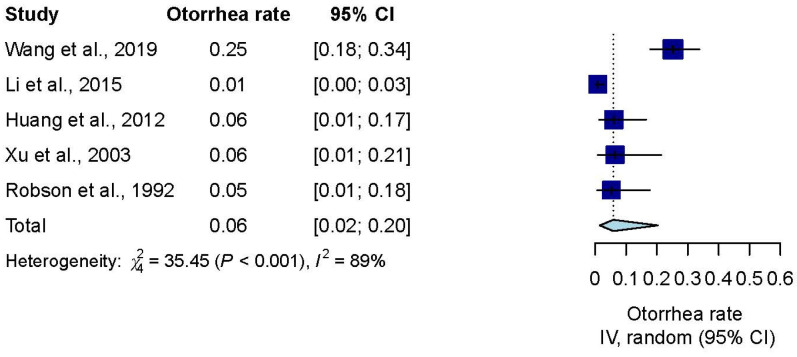
Effect estimate of post-VTI otorrhea rate.

Five studies [29,30,31,33,37] that reported post-VTI otorrhea data were pooled for effect estimation. The pooled otorrhea rate was 6% (rate, 0.06; 95% CI, 0.02–0.2; *I*^2^ = 89%) (Figure 4).

### 3.4. Influence Analysis

**Figure 5:**
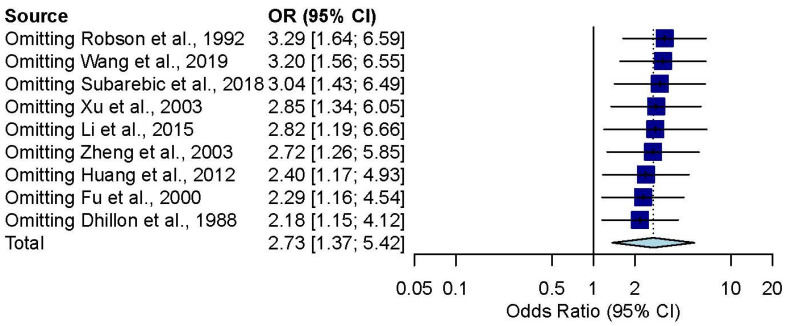
Influence analysis of the overall effect of palatal repair with VTI.

In the influence analysis, the pooled point estimates after excluding every study one by one were contained within the 95% CI of the overall pooled results for these outcomes (Figure 5).

### 3.5. TSA

**Figure 6:**
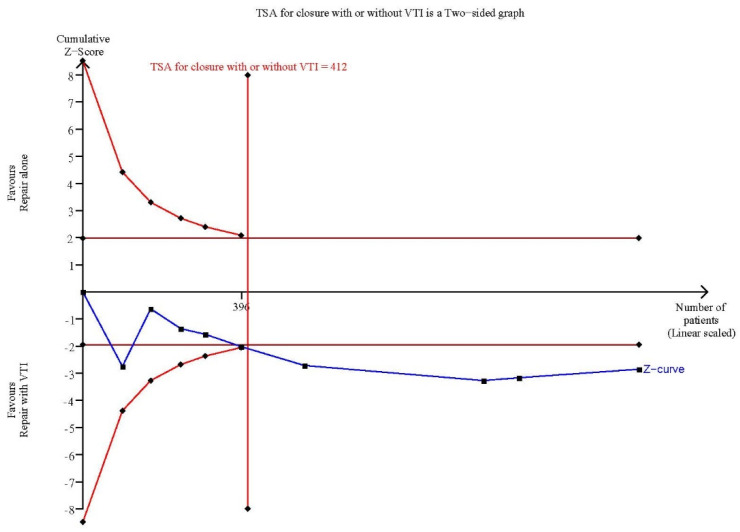
Trial sequential analysis (TSA) of overall effect of palatal repair with VTI.

**Figure 7:**
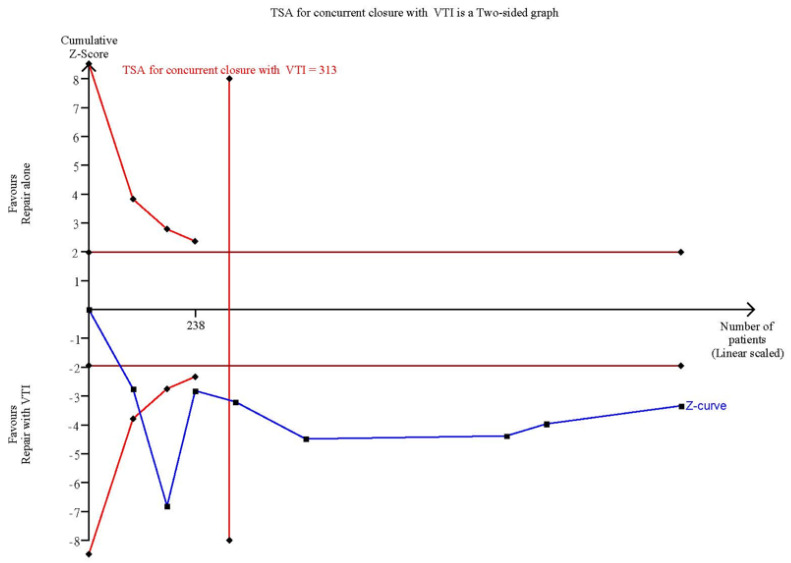
TSA of effect of concurrent VTI with palatal repair.

The cumulative Z-curves surpassed both the traditional significance boundary and the sequential monitoring boundaries for the adjusted significance threshold in favor of palatal repair with VTI after the required information size (RIS) was reached in the TSA of the overall analysis (Figure 6) and the subgroup analysis of concurrent VTI with palatal repair (Figure 7). Consequently, the TSAs indicated conclusive results for these outcomes.

### 3.6. Publication Bias

**Figure 8:**
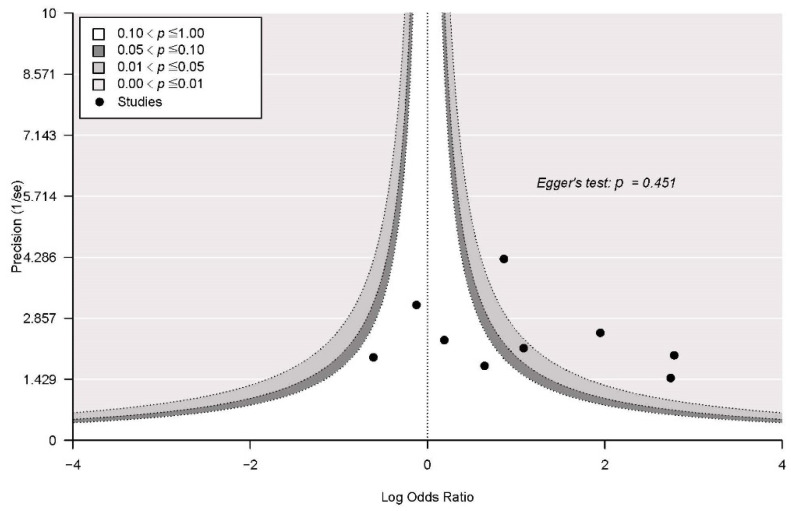
Funnel plots with Egger’s test for publication bias.

Publication bias was assessed for the overall results with contour-enhanced funnel plots. Egger’s test revealed no significant asymmetry in either outcome (*p* = 0.466) for the overall assessment (Figure 8).

## 4. Discussion

The paramount finding of the present study is that surgical palatal repair accompanied by VTI in the middle ear could significantly prevent patients with cleft palate from developing OME. Further subgroup analysis indicated that the benefit of VTI could be sustained when the ventilation tube was placed during the palatal repair operation. On the other hand, placing the ventilation tube after palatal repair surgery did not show benefit for preventing OME development. Subsequent TSA provided further conclusive results regarding the overall analysis and subgroup analysis. Additionally, a meta-analysis of the post-VTI otorrhea rate revealed that approximately 6% of patients might develop post-VTI otorrhea.

Issues regarding VTI in patients with cleft palate have been discussed for decades [10,36,38,39]. In a previous review [10] of patients with cleft palate, the efficacy of palatal repair with VTI was greater than that of palatal repair without VTI, as the rate of OME development was 15% to 20% for palatal repair with VTI and 56% to 80% for palatal repair without VTI. However, no quantitative evidence is currently available. Therefore, we systematically examined all relevant studies and obtained quantitative results via meta-analysis. Subsequent TSA further supported the reliability of the results. To our knowledge, this is the first meta-analysis to provide quantitative evidence on the efficacy of VTI with palatal repair in patients with cleft palate.

Patients with cleft palate often suffer from middle ear dysfunction and hearing loss, which may result from the abnormal function of the eustachian tube in patients with cleft palate; the eustachian tube cannot be opened by nonfunctional muscular structures (i.e., tensor veli palatini and levator veli palatini) [3,40]. This pathology of OME in patients with cleft palate is considered to be associated with immune and inflammatory reactions that may result from rhinopharyngeal infection or irritation with regurgitated food or fluid via the palatal defect. Following cytokine production, exudates with inflammatory mediators play a key role as vasodilators in the middle ear. The subsequent vasodilatation results in gaseous exchanges, which causes an endotympanic pressure drop in the middle ear [9,41,42]. The negative pressure in the middle ear then further produces retraction of the tympanic membrane. For those with cleft palate, the dysfunction of the eustachian tube further deteriorates the situation [19,43]. The exudate cannot be cleared through the eustachian tube and is persistently trapped in the middle ear cavity, leading to the development of OME. Additionally, the locally resident flora (e.g., Streptococcus pneumoniae, Haemophilus influenza) may simultaneously proliferate under the favorable conditions, leading to a vicious cycle of inflammation and structural dysfunction, and in turn, intractable OME [42,43]. As the OME worsens, the condition becomes more complicated. The retractive tympanic membrane progresses to perforation of the eardrum, chronic otitis media and even cholesteatoma, which may be further complicated by deformities of ossicles and even osteomyelitis. Consequently, irreversible sequalae such as hearing loss or facial palsy may develop [43]. According to previous studies, approximately 90% of patients with cleft palate are diagnosed with OME at birth, and approximately 2% to 24% of patients with cleft palate develop long-term hearing loss; this rate may even reach 50% when there is insufficient knowledge of cleft palate. As a result, patients may suffer from deteriorating quality of life with a profound influence on their speech ability owing to subsequent hearing loss, which could eventually lead to learning disabilities and cause psychosocial problems, including withdrawal from society, poor self-esteem or even depression. The increase in the cost of social care could be considerable, as the cost for the supportive care of those with hearing loss ranges from USD5075 to USD13,731 according to the World Health Organization (WHO) [44]. Under these circumstances, the management of OME in patients with cleft palate is important.

Significant breakthroughs have been made in the treatment of cleft palate in recent decades. However, the tendency to develop OME is not totally reversed by palatal repair surgery [3,9]. Consequently, VTI has been deemed a potentially effective procedure for persistent OME in patients with cleft palate. After the repair of cleft palate, inserting a ventilation tube can diminish the negative pressure in the middle-ear cavity by replacing the absorbed gases by the vasodilation in the middle ear, and the trapped effusion can be drained out via the functioning Eustachian tube to break the vicious cycle of inflammation and structural dysfunction described above [3,13,14]. The efficacy of VTI has been proven not only for patients with cleft palate but also for those with normal variants who have persistent OME [42,45,46]. These patients are usually children with hypertrophic nasopharyngeal adenoid, and standalone adenoidectomy could only achieve normalization of afunctional Eustachian tube. Elimination of negative pressure in middle ear still relies on the gases exchanged via the inserted tube [47]. However, debates regarding routine VTI with palatal repair remain, partly due to the uncertainty of the timing of insertion and concerns regarding complications of VTI [3,19,42]. In addition to the objective evidence of the efficacy of VTI for patients with cleft palate provided by the primary analysis, further subgroup analysis suggested that concurrent VTI with palatal repair would have benefitted most patients who underwent elective VTI after palatal repair. Previous studies have illustrated the long-term alterations of the middle-ear structures and environment in those who suffer from OME. As OME worsens, the disease becomes more complicated and progresses from a depression in the eardrum to perforation of the eardrum, chronic otitis media, and finally even cholesteatoma, accompanied by loss of ossicles and even osteomyelitis [48,49]. Patients undergoing elective VTI after palatal repair may experience substantial changes in the microenvironment of the middle-ear cavity before tube placement and therefore receive less benefit from VTI [49,50,51,52,53]. Further direct evidence regarding the middle-ear condition should be presented, and we suggest more studies regarding this aspect in the future.

Post-VTI otorrhea is regarded as the most common complication of tympanostomy tube placement. The incidence of otorrhea after the procedure varies from 3.4% to 74% [54]. In the present study, we reported an incidence of 6% (95% CI, 2–20%) of post-VTI otorrhea, which seems reasonable according to previous data. As the ventilation tube causes direct communication between the middle-ear cavity and external ear, otorrhea, which largely results from the inflammation of the eustachian tube and the middle ear due to the common cold, is inevitable. Fortunately, most cases of otorrhea are simple and manageable using ototopical drips or water precautions. Unmanageable cases that require removal of the tube are relatively rare. Under these circumstances, it is important to appropriately explain the risk of otorrhea to the patients or the family of patients before the operation [54,55]. On the other hand, reasonable selection of the ventilation tube also plays a key role to prevent post-VTI otorrhea. Biocompatibility and surface composition are two important components to be considered for tube insertion [16]. Metal ventilation tubes, including gold tubes and titanium tubes, have been proven less biocompatible when compared to plastic tubes, including silicone or fluoroplastic tubes, and were not suitable for long-term use [17,18]. Furthermore, plastic ventilation tubes are frequently coated with various substances, including silver oxide, high energy argon atoms, or even antibiotics. These coating materials could further lower the incidence of infection and biofilm formation on the tubes, which would be applied to patients with risk of middle-ear infection. The majority of included studies adopted silicone ventilation tubes for intervention, and this may explain the reasonable post-VTI otorrhea rate of our study. We suggest that clinicians should also take into account patients’ clinical features and the expected duration of tube placement when performing VTI.

There are limitations to our work. First, most of the included studies did not clarify the method of allocation to the experimental and control groups. For an interventional study, the best research design is a randomized controlled trial (RCT). However, the included studies did not state the allocation method or mention the study design. To avoid systematic bias, we used a relatively conservative tool for risk assessment, the ROBINS-I tool, to evaluate the methodological quality of the included studies. Second, most of the included studies did not provide age data in detail. Although we used subgroup analysis to successfully show that concurrent VTI with palatal repair is more beneficial, the best time point for the operation relies on analyzing the correlation between age and effect estimate by meta-regression. Third, there are some potential factors that would cause heterogeneity, including the ambiguity of allocation, the method of outcome evaluation, difference of age and follow-up length across the included studies. To account for the expected heterogeneity that cannot be calculated and quantified, we chose the random-effects model instead of the fixed-effect model and performed influence analysis. However, no obvious outlier was noted. Fourth, although the subgroup analysis and trial sequential analysis indicated that the efficacy of concurrent VTI with palatal repair was significant, the number of studies regarding non-concurrent VTI was insufficient for subgroup analysis and trial sequential analysis. The efficacy of non-concurrent VTI remained uncertain in the present study. Finally, the present study only analyzed otorrhea as a complication. The complications of VTI are quite variable, including postoperative infection and tube-related problems (e.g., displacement, persistent perforation), and only data on post-VTI otorrhea from the included studies were sufficient for analysis. As a result, we suggest that more studies provide comprehensive data in the future.

## 5. Conclusions

We evaluated the efficacy of VTI for OME elimination in patients with cleft palate undergoing palatal repair. The overall result suggested that VTI is effective for eliminating OME in patients with cleft palate. Further subgroup analysis indicated that the benefit of VTI is significant if the palatal repair was performed concurrently. Evidence regarding non-concurrent VTI with palatal repair for prevention of OME remained insufficient, and further large-scale trials are essential for conclusive results.

## Figures and Tables

**Table 1:** Study characteristics.

Study	Region	Intervention	Control	Patient	Sample Size	Outcome of Intervention	Outcome of Control	Age	VTI Timing	Outcome Evaluation	Tube Material	Post-VTI Otorrhea Rate
Wang et al., 2019 [29]	Asia	Palatal repair plus VTI	Palatal repair only	155 *	298	75/242	19/56	11.59 m	With palatal repair	Tympanography andOtoscopy	Silicone	31/123
Li et al., 2015 [30]	Asia	Palatal repair plus VTI	Palatal repair + tympanocentesis	274 *	446	210/248	139/198	5.7 years	With palatal repair	Tympanogram	Silicone	2/248
Huang et al., 2012 [31]	Asia	Palatal repair plus VTI	Palatal repair only	99 *	158	41/78	11/80	1–7 years	With palatal repair	Tympanogram	Silicone	3/50
Zheng et al., 2003 [32]	Asia	Palatal repair plus VTI	Palatal repair only	62 *	88	19/39	12/49	4.68 years	With palatal repair	Tympanogram	Silicone	NR
Xu et al., 2003 [33]	Asia	Palatal repair plus VTI	Palatal repair only	53 *	62	11/31	7/31	0.5–8 years	With palatal repair	Tympanogram	NR	2/31
Fu et al., 2000 [34]	Asia	Palatal repair plus VTI	Palatal repair only	76	76	28/45	3/31	3–16 years	With palatal repair	Tympanogram	Silicone	NR
Robson et al., 1992 [37]	Europe	Palatal repair plus VTI	Palatal repair only	70	70	22/38	23/32	5.8 years	After palatal repair	Tympanogram	NR	2/38
Subarebic et al., 2018 [35]	Europe	Palatal repair plus VTI	Palatal repair only	90	90	21/45	19/45	1–6 years	With palatal repair	Tympanogram and microscopy	NR	NR
Dhillon et al., 1988 [36]	Europe	Palatal repair plus VTI	Palatal repair + tympanocentesis	50 *	100	40/50	10/50	11.5 years	With palatal repair	Tympanogram and otoscopy	Silicone	NR

The asterisk (*) indicates studies that performed analysis by using ears as a unit of measurement; VTI: Ventilation tube insertion; NR: Not reported.

## Data Availability

Not applicable.

## Supplementary Materials

The following supporting information can be downloaded at: https://www.mdpi.com/article/10.3390/jpm12020255/s1, Figure S1: Risk of Bias; Figure S2: Summary of Risk of Bias; Table S1: Detailed search strategy.
