# Supplementary material for: Efficacy of Ventilation Tube Insertion with Palatal Repair for Otitis Media in Cleft Palate: Meta-Analysis and Trial Sequential Analysis
Source: J Pers Med. 2022 Feb 10;12(2):255. doi: 10.3390/jpm12020255 (PMC8875192; doi:10.3390/jpm12020255)

Supplemental Materials

Efficacy of Ventilation Tube Insertion with Palatal Repair for Otitis Media in Cleft Palate: Meta-Analysis and Trial Sequential Analysis

Feng-Liang Chang, Chih-Hao Chen, Hsiu-Lien Cheng, Chun-Yu Chang, Jing-Li Leong, Yen-Ting Chang, Yen-Fu Cheng and Wen-Huei Liao

Table S1. Detailed search strategy

Database	Query
PubMed	((("Cleft Palate"[Mesh]) OR ("Cleft Palate/analysis"[Mesh] OR "Cleft Palate/anatomy and histology"[Mesh] OR "Cleft Palate/classification"[Mesh] OR "Cleft Palate/congenital"[Mesh] OR "Cleft Palate/diagnosis"[Mesh] OR "Cleft Palate/epidemiology"[Mesh] OR "Cleft Palate/etiology"[Mesh] OR "Cleft Palate/genetics"[Mesh] OR "Cleft Palate/prevention and control"[Mesh] OR "Cleft Palate/surgery"[Mesh] OR "Cleft Palate/therapy"[Mesh])) OR "Cleft"[TIAB] OR "Cleft Palates"[TIAB] OR "Cleft Palate"[TIAB] OR "Cleft Palate, Isolated"[TIAB] OR "Isolated cleft palate"[TIAB] OR "Syndromic cleft palate"[TIAB] OR "Cleft" OR "Cleft Palates" OR "Cleft Palate" OR "Cleft Palate, Isolated" OR "Isolated cleft palate" OR "Syndromic cleft palate") AND (("Middle Ear Ventilation"[Mesh]) OR ("Middle Ear Ventilation/instrumentation"[Mesh] OR "Middle Ear Ventilation/methods"[Mesh] OR "Middle Ear Ventilation/standards"[Mesh] OR "Middle Ear Ventilation/therapeutic use"[Mesh] OR "Middle Ear Ventilation/therapy"[Mesh]) OR "Ventilation tube"[TIAB] OR "Middle Ear Ventilation"[TIAB] OR "Grommet Insertion"[TIAB] OR "Grommet Insertions"[TIAB] OR "Tympanostomy Tube Insertion"[TIAB] OR "Tympanostomy Tube Insertions"[TIAB] OR "Ventilation tube" OR "Middle Ear Ventilation" OR "Grommet Insertion" OR "Grommet Insertions" OR "Tympanostomy Tube Insertion" OR "Tympanostomy Tube Insertions") AND (((("Otitis Media with Effusion"[Mesh]) OR ("Otitis Media with Effusion/complications"[Mesh] OR "Otitis Media with Effusion/prevention and control"[Mesh] OR "Otitis Media with Effusion/therapy"[Mesh])) OR "Hearing Loss"[Mesh]) OR ("Hearing Loss/complications"[Mesh] OR "Hearing Loss/therapy"[Mesh]) OR "OME"[TIAB] OR "Middle Ear Effusion"[TIAB] OR "Middle Ear Effusions"[TIAB] OR "Secretory Otitis Media"[TIAB] OR "Serous Otitis Media"[TIAB] OR "Hearing loss"[TIAB] OR "Hypoacusis"[TIAB] OR "Hearing Impairment"[TIAB] OR "Transitory Deafness"[TIAB] OR "Transitory Hearing Loss"[TIAB] OR "OME" OR "Middle Ear Effusion" OR "Middle Ear Effusions" OR "Secretory Otitis Media" OR "Serous Otitis Media" OR "Hearing loss" OR "Hypoacusis" OR "Hearing Impairment" OR "Transitory Deafness" OR "Transitory Hearing Loss")
Embase	('cleft palate'/exp OR cleft OR 'cleft palates' OR 'cleft palate, isolated' OR 'isolated cleft palate' OR 'syndromic cleft palate') AND ('ventilation tube'/exp OR 'middle ear ventilation'/exp OR 'grommet insertion' OR 'grommet insertions' OR 'tympanostomy tube insertion' OR 'tympanostomy tube insertions') AND (ome OR 'middle ear effusion' OR 'middle ear effusions' OR 'secretory otitis media'/exp OR 'serous otitis media'/exp OR 'hearing loss' OR hypoacusis OR 'hearing impairment'/exp OR 'transitory deafness' OR 'transitory hearing loss')
Web of Science	TS=(("Cleft" OR "Cleft Palates" OR "Cleft Palate" OR "Cleft Palate, Isolated" OR "Isolated cleft palate" OR "Syndromic cleft palate")AND("Ventilation tube" OR "Middle Ear Ventilation" OR "Grommet Insertion" OR "Grommet Insertions" OR "Tympanostomy Tube Insertion" OR "Tympanostomy Tube Insertions")AND("OME" OR "Middle Ear Effusion" OR "Middle Ear

	Effusions" OR "Secretory Otitis Media" OR "Serous Otitis Media" OR "Hearing loss" OR "Hypoacusis" OR "Hearing Impairment" OR "Transitory Deafness" OR "Transitory Hearing Loss"))
Scopus	(TITLE-ABS-KEY("Cleft" OR "Cleft Palates" OR "Cleft Palate" OR "Cleft Palate, Isolated" OR "Isolated cleft palate" OR "Syndromic cleft palate") AND TITLE-ABS-KEY("Ventilation tube" OR "Middle Ear Ventilation" OR "Grommet Insertion" OR "Grommet Insertions" OR "Tympanostomy Tube Insertion" OR "Tympanostomy Tube Insertions") AND TITLE-ABS-KEY("OME" OR "Middle Ear Effusion" OR "Middle Ear Effusions" OR "Secretory Otitis Media" OR "Serous Otitis Media" OR "Hearing loss" OR "Hypoacusis" OR "Hearing Impairment" OR "Transitory Deafness" OR "Transitory Hearing Loss"))
Cochrane Library	ID Search<br>#1 MeSH descriptor: [Cleft Palate] explode all trees<br>#2 MeSH descriptor: [Cleft Palate] explode all trees and with qualifier(s): [classification - CL, diagnosis - DI, etiology - ET, genetics - GE, epidemiology - EP, prevention & control - PC, surgery - SU, therapy - TH]<br>#3 cleft<br>#4 cleft palates<br>#5 cleft palate<br>#6 Cleft Palate, Isolated<br>#7 Isolated cleft palate<br>#8 Syndromic cleft palate<br>#9 {OR #1-#8}<br>#10 MeSH descriptor: [Middle Ear Ventilation] explode all trees<br>#11 MeSH descriptor: [Middle Ear Ventilation] explode all trees and with qualifier(s): [instrumentation - IS, methods - MT, standards - ST]<br>#12 Ventilation tube<br>#13 Middle Ear Ventilation<br>#14 Grommet Insertion<br>#15 Grommet Insertions<br>#16 Tympanostomy Tube Insertion<br>#17 Tympanostomy Tube Insertions<br>#18 {OR #10-#17}<br>#19 MeSH descriptor: [Otitis Media with Effusion] explode all trees<br>#20 MeSH descriptor: [Otitis Media with Effusion] explode all trees and with qualifier(s): [complications - CO, prevention & control - PC, therapy - TH]<br>#21 MeSH descriptor: [Hearing Loss] explode all trees<br>#22 MeSH descriptor: [Hearing Loss] explode all trees and with qualifier(s): [complications - CO, therapy - TH]

	#23 MeSH descriptor: [Speech] explode all trees<br>#24 MeSH descriptor: [Cholesteatoma] explode all trees<br>#25 MeSH descriptor: [Cholesteatoma] explode all trees and with qualifier(s): [complications - CO, surgery - SU, therapy - TH]<br>#26 MeSH descriptor: [Postoperative Complications] explode all trees<br>#27 OME<br>#28 Middle Ear Effusion<br>#29 Middle Ear Effusions<br>#30 Secretory Otitis Media<br>#31 Serous Otitis Media<br>#32 Hearing loss<br>#33 Hypoacusis<br>#34 Hearing Impairment<br>#35 Transitory Deafness<br>#36 Transitory Hearing Loss<br>#37 speech<br>#38 public speaking<br>#39 cholesteatoma<br>#40 cholesteatomas<br>#41 postoperative complication<br>#42 {OR #19-#36}<br>#43 #9 AND #18 AND # 42

Figure S1. Risk of Bias. The Risk of Bias in Non-randomized Studies - of Interventions (ROBINS-I) was used to evaluate to quality of included studies.

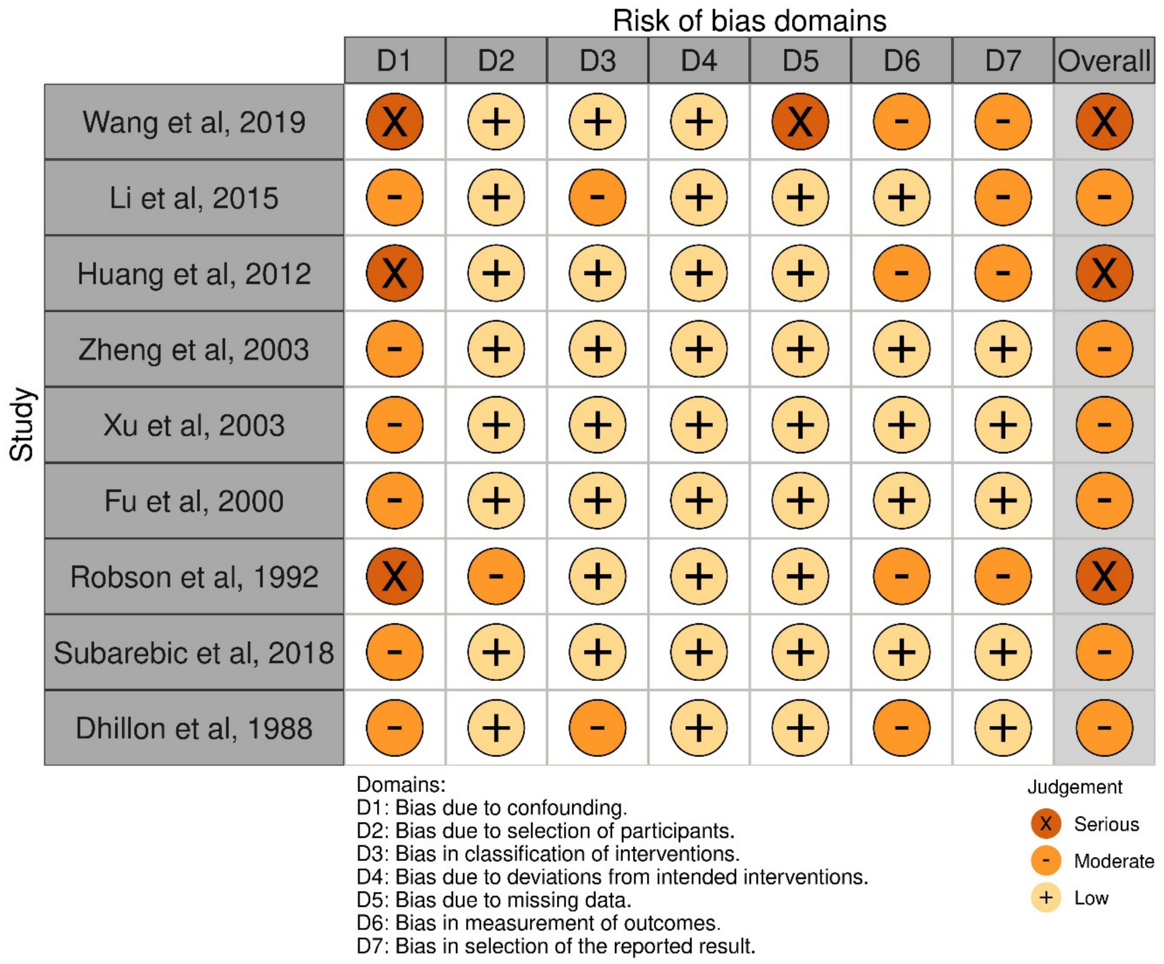

Figure S2. Summary of Risk of Bias. Overall, 67% of the studies are subject to moderate risk of bias while another 33% are subject to serious risk of bias. Sixty-six percents of studies have moderate risk in bias due to confounding, while 33% of studies may sustain serious risk in bias due to confounding. 11% of studies have moderated risk of bias due to selection of participnats. 22% of studies sustain moderate risk in classification of interventions. 11% of studies have moderated risk of bias due to missing data. 44% of studies have moderate risk in bias in meaurement of outcomes. 44% of studies have moderate risk in bias in selection of the reported result.

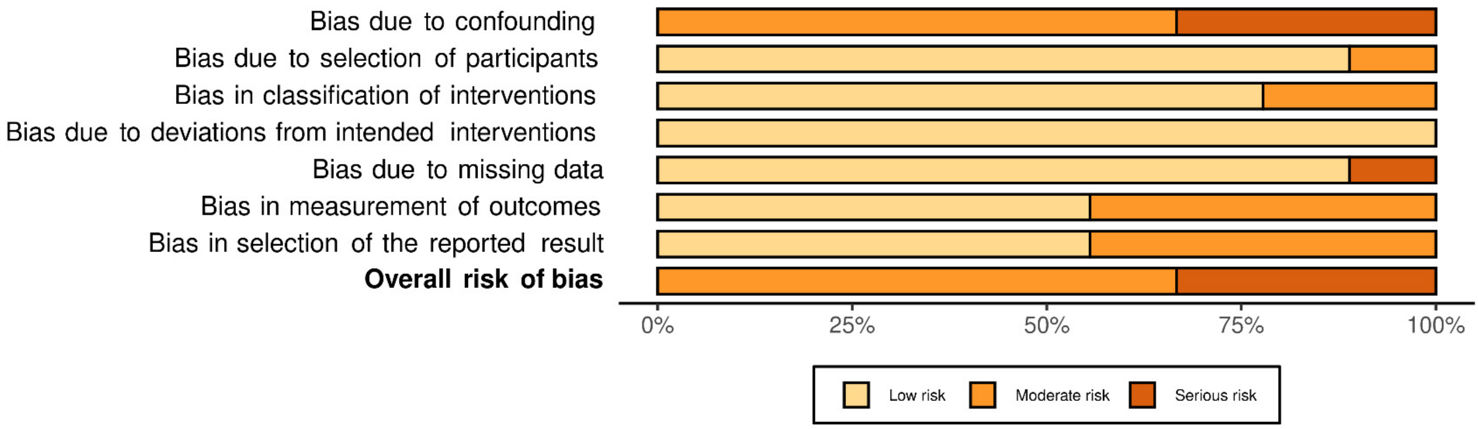

Supplement: Supplementary file 1 [file jpm-12-00255-s001.zip › jpm-1583963-SI.pdf]
